# Printed Sensors for Quantifying Electrodermal Activity and Sweat Rate: A Review

**DOI:** 10.3390/s25226878

**Published:** 2025-11-11

**Authors:** Batoul Hosseinzadeh, Sarah Tonello, Nicola Francesco Lopomo, Emilio Sardini

**Affiliations:** 1 Department of Information Engineering, University of Brescia, Via Branze, 38, 25123 Brescia, Italy; sarah.tonello@unibs.it (S.T.); emilio.sardini@unibs.it (E.S.); 2 Department of Design, Politecnico di Milano, Piazza Leonardo da Vinci 32, 20133 Milano, Italy

**Keywords:** electrodermal activity, printed sensors, sweat monitoring

## Abstract

Monitoring electrodermal activity (EDA) and sweat rate (SR) and volume hold promise for yielding neurological health insights about individuals. A combination of standard EDA monitoring with the quantitative analysis of perspired sweat volume, rate, and composition represents a promising advancement for improving the understanding and reliability of EDA signals. In this picture, exploiting printed electronics to face challenges related to bulky gold-standard setups and to achieve integration in fully wearable devices represents one of the most interesting approaches addressed by recent research. In this review, we present an overview of the principal techniques, materials, and measurement methods reported for fabricating EDA and sweat monitoring electrodes. We highlight the increasing effect of printing technologies as a key enabler for scalable, low-cost, and customizable fabrication of flexible sensors suited for on-skin applications. These approaches not only support mass production but also enhance adaptability and comfort in wearable formats. Overall, the review emphasizes how printed technologies significantly improve physiological signal quality and open new opportunities for continuous, non-invasive, and personalized health monitoring.

## 1. Introduction

Very recent developments in smart wearable systems have been significantly extending healthcare services beyond the confines of the mere clinical environment, providing essential and timely information on overall health conditions, no matter where the subjects are located [1,2]. Despite significant improvement in wearables observed over the past few decades, including commercialized examples such as smartwatches, sport bands, etc., substantial challenges and opportunities for advancements are still open; in particular, further progress is required to extend applicability, comfort, metrological accuracy, and robustness of these solutions [3]. In addition to the widespread interest in traditional physiological signals (e.g., heart rate, body temperature, blood oxygen levels, physical activity [4,5,6]), researchers have been recently focusing on developing non-invasive, lightweight, and minimally obtrusive devices (e.g., patches, tattoos) to investigate skin properties, which serve as relevant indicators for more comprehensive insights into health conditions. Measuring skin properties encompasses the monitoring of both active and passive skin electrical characteristics (e.g., conductivity, impedance) [7]; these processes are generally indicated as measurements of EDA or Galvanic Skin Response (GSR). Furthermore, quantifying the volume, rate, and chemical composition of the sweat accumulated on the skin surface represents a key activity in the estimation of overall health conditions [8].

Monitoring EDA shows significant potential for providing comprehensive insights into overall health [9]. This is because EDA is closely linked to several vital physiological parameters, including changes in blood pressure, increased heartbeat, and perspiration. These responses occur specifically when stimuli activate the Sympathetic Nervous System (SNS); in fact, the SNS is a key component of the Autonomic Nervous System (ANS), which is directly involved in regulating psychological, cognitive, and emotional arousal, as well as stress responses [10,11]. A typical EDA signal is acquired by placing two adhesive pre-gelled electrodes on the skin surface of areas with the highest concentration of sudoriferous glands (e.g., hand fingers, palm) and measuring electrical conductance, resistance, impedance, or admittance of the skin, depending on the specific acquisition method that can be employed. Despite EDA being a quite simple and established technique, several challenges still need to be faced to enable its broad, continuous, reliable, and time-extensive evaluation. The main issue to solve is represented by the use of bulky electrodes, which are traditionally employed in most measurement setups. Achieving affordable, small, and inobtrusive sensors could provide the comfort required to ensure user acceptability and overall usability. A critical factor to consider is that the volume and composition of sweat on the skin significantly affects the EDA signal. In this regard, the combination of standard EDA electrodes with a sensing patch providing feedback on sweat volume and composition represents an interesting advancement that could help to improve the overall reliability of EDA measurements.

If combined with EDA monitoring, real-time monitoring of sweat flow offers a valuable and deeper understanding of psychophysical stress as well as physical conditions, including also hyperhidrosis or hypohidrosis [12]. In fact, sweating is a crucial physiological reaction for dissipating heat and regulating body temperature under heat-stress conditions. In sport and exercise medicine (SEM), monitoring SR assists athletes in evaluating their dehydration levels, allowing them to rehydrate appropriately. Furthermore, individual sweat flow profiles can represent signs of dysautonomia, a common sign in ischemic stroke patients [13]. Beyond the volumetric sweat quantification, a more specific analysis of its rich composition in terms of ions and metabolites holds significant potential. The current research focused on highlighting possible relations between sweat composition and health indicators, such as blood sugar, levodopa profiles, and dehydration levels, has been supporting the need for individualized wearable sweat sensors [14]. Moreover—in contrast with gravimetric testing techniques, which are generally employed to measure total sweat loss—wearable devices offer the possibility to realize real-time measurements addressing increased portability and user-friendliness [15].

Furthermore, printed electronics are rapidly evolving, offering flexible, lightweight, and cost-effective alternatives to traditional fabrication methods. These advancements enable the development of high-performance wearable sensors with improved conformability to the skin, enhancing user comfort and measurement accuracy. In particular, printed sensors for EDA and sweat monitoring play a crucial role in achieving reliable, real-time physiological tracking. By leveraging scalable manufacturing processes and biocompatible materials, printed electronics have been contributing to the development of next-generation wearables that seamlessly integrate into daily life, supporting health monitoring and personalized medicine.

**Figure 1:**
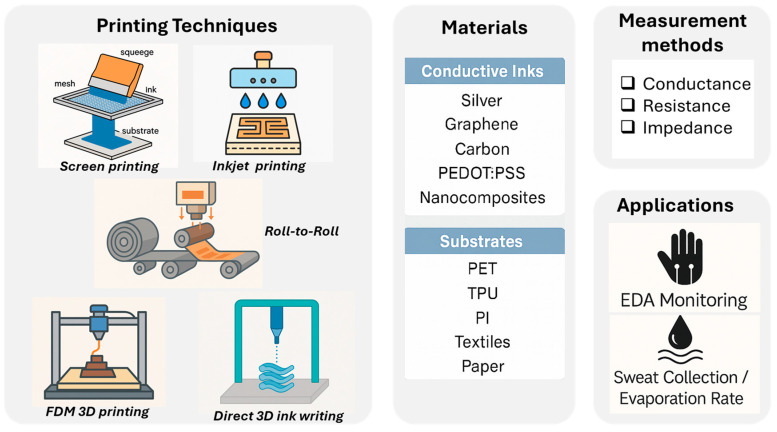
Overview of various printing techniques with employed materials and applications.

To date, it is worth noting that several comprehensive reviews have been published on wearable sweat sensors, specifically addressing aspects such as materials and complex analytical frameworks. Notably, the work by Min et al. is highly relevant as it offers a deep analysis of the innovations and challenges related to the development of sweat-interfaced wearables for precision medicine applications [16]. Another contribution is given by Heikenfeld et al. [17], who focused on wearable sweat sensors while exploring the most recent updates and problems in the implementation of wearable solutions for physiological fluidics and biomarker quantification. Finally, Ghaffari et al. explored the integration of multi-sensing wearables with soft microfluidics [18]. While all these reviews have advanced the understanding of wearable sensing, mainly focusing on biomarkers, sweat-rate, fluid volume, and EDA, they still neglect the engineering and metrological challenges specific to printed transduction systems. Starting from the most recent advancements in the field of EDA and sweat monitoring [19,20], this review aims to consolidate current knowledge on printing technologies enabling scalable and multimodal sweat monitoring. Specifically, this work critically analyzes the state-of-the-art of printed sensors designed to measure EDA and/or sweat volume or rate. Particular attention is given to fabrication processes, functional materials, flexible and stretchable substrates, and microfluidic sweat collection strategies. Moreover, the main measurement methods are discussed, highlighting the metrological parameters commonly used to compare sensor performance. Figure 1 schematically summarizes the manufacturing techniques, materials, and measurement methods identified for the preparation of the printed sensors and their application.

## 2. Sources, Search Methods, and Study Selection

The review took into consideration original studies identified on the Scopus, Pubmed, and Web of Science databases and published in the last decade, from 2015 to 2024. The search was performed on the 17 October 2025, searching for titles, abstracts, and keywords by using the following string:

TITLE-ABS-KEY (“printed” OR “deposited” OR “additively manufactured”) AND TITLE-ABS-KEY (sensor* OR electrode*) AND TITLE-ABS-KEY (“electrodermal activity” OR “galvanic skin response” OR “EDA” OR “skin conductance” OR “perspiration” OR “sweat rate”) AND PUBYEAR > 2014 AND PUBYEAR < 2026.

In order to track the current trends in the scientific literature, we decided to also include conference papers; on the other hand, review papers were excluded. Articles selected for this review study were included only if they met the following criteria:

- Proposing a sensor for either EDA or SR/volume quantification;
- Exploiting a printed electronics technique for sensor fabrication;
- Providing sufficient information about the fabrication process for electrode realization and integration with either customized or commercial electronics.

The first screening round was performed on titles/abstracts to exclude all papers focused on topics other than SR/volume quantification or EDA measurement. Two reviewers were involved in the inclusion/exclusion process, specifically following the previously reported criteria, whereas a third reviewer supported the assessment in case of discrepancy.

Screened papers were then exported and analyzed, and details regarding fabrication processes of the electrodes, sweat collection modes, and measurement parameters were extracted, compared, discussed through the text, and summarized in the cumulative tables reported at the end of the paper. Particular attention was given to the following:

- Application and details of the printing process, including inks, substrate, flexibility and stretchability, and electrode geometry (Table 1, Table 2 and Table 3);
- Details on the sweat collection methods (material, geometry, fabrication technique) (Table 4);
- Measurement method details (transduction methods, conditioning electronics details) (Table 5).

**Figure 2:**
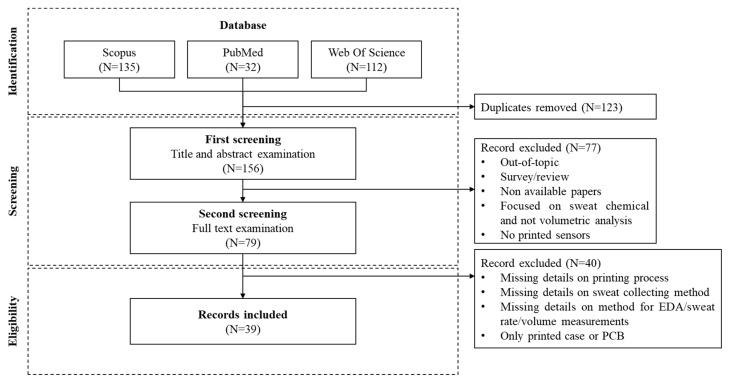
PRISMA diagram for review screening.

The initial literature search identified 80 papers (duplicates excluded) according to the string used as reported above. The screening was performed following the PRISMA algorithm as depicted in Figure 2. Finally, 26 manuscripts were finally identified as the most representative to discuss. Based on in-depth analysis of those papers, the following sections address the most relevant standard parameters for validation, the validation procedures, and the electronic design considerations. Finally, an overall discussion is provided on the main opportunities and challenges highlighted in the comparative analysis.

## 3. Fabrication Techniques

The variety of printing methods that were identified to be effectively used to realize EDA and SR sensors were classified into contact and non-contact (or nozzle-based) printing techniques [21]. Furthermore, properly defined 3D printing techniques were considered as an additional category to consider, since they were often used to create custom molds or substrates for subsequent electrode fabrication, rather than directly printing functional electrodes.

### 3.1. Contact Printing

Contact printing techniques represent the most frequently employed approach for manufacturing EDA and SR electrodes, when targeting simple and macroscopic geometries with high throughput and fast printing [22]. This approach includes all those methods that exploit plates or rolls that directly touch the substrate, applying external pressure to enhance ink adhesion and penetration into the substrate, making it ideal for printing on large-size areas [23]. The techniques in this category, as identified in the analyzed literature, include screen printing, roll-to-roll printing, and gravure printing. A general overview of the identified solutions is reported in Table 1. Although Table 1 includes predominantly screen-printed sensors, this reflects the current state of the field, as roll-to-roll and gravure printing have been less frequently reported for EDA and sweat rate applications. These methods, however, offer promising scalability for development.

**Table 1:** Contact printing techniques-based EDA and sweat monitoring electrodes.

Printing Technique	Inks	Substrate	Flexibility/Stretchability	Electrode Geometry	Application	Refs
Screen printing	silver	TPU	flexible and stretchable	circular (6 and 8 mm)	multi-purpose (ECG, EDA, acceleration, temperature, humidity)	[24]
	silver	polyimide	flexible	customized plasmonic structure (2 mm)	sweat sensing	[25]
	graphene-doped carbon paste	polyethylene terephthalate (PET)	flexible	interdigitated (dimensions not specified)	sweat sensing	[26]
	silver + carbon	TPU	flexible and stretchable	rectangular (from 0.5 to 8 mm)	sweat sensing	[27]
	carbon	polyimide	flexible	interdigitated (3 mm teeth)	sweat sensing	[28]
	PEDOT:PSS	nonwoven PET	flexible and stretchable	rectangular (2 × 3 cm)	multi-purpose (ECG, EDA, EMG)	[29]
	silver	PLA	flexible and stretchable	circular (5 mm)	EDA	[30]
	carbon	PET	flexible	circular and interdigitated	EDA and sweat analysis/skin condition	[31]
	PEDOT, PVDF-TrFE ink and Ag	PDMS	flexible and stretchable	matrix of circular electrodes	electronic skin (EDA combined with EMG and motion tracking)	[32]
Roll-to-Roll	silver	PET	not flexible	comb-like in spiral structure (thickness of electrodes: 0.1 mm)	SR	[33]
	silver	polymeric	flexible	various geometries	sweat rate and volume	[34]
	silver and graphite inks	dielectric polymer layers; PET microfluidic cover;	flexible multilayer patch	two spirals	sweat rate and composition (Na^+^, K^+^, glucose, etc.)	[35]
Gravure printing	silver	polycarbonate polyethylene glycol terephthalate	flexible and stretchable	square (2 mm)	EDA	[36]
	carbon/silver/catalyst/metal-based functional inks.	PET	flexible	circular, traces, micro-electrode arrays	sweat sensing	[37]

**Figure 3:**
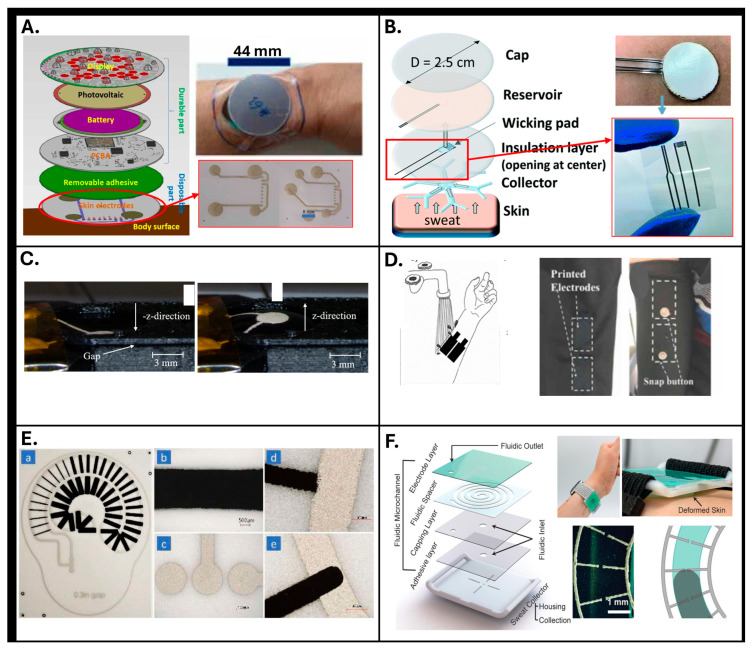
Examples of EDA and SR electrodes fabricated with contact-based techniques: images (**A**–**F**) are screen-printed electrodes. In detail: (**A**) circular electrodes inserted in a vertically aligned structure to realize a stand-alone multi-purpose patch [24], copyright 2023 IOP; (**B**) interdigitated electrodes integrated with a bio-inspired collecting-paper-based microfluidics [26], copyright 2021 Royal Society of Chemistry; (**C**) circular EDA electrodes printed on top of a deformable membrane [30], copyright 2017 MDPI; (**D**) rectangular-shaped electrodes based on a biodegradable polymer, PEDOT:PSS printed on textile [29], copyright 2020 IOP; (**E**) customized geometry with multiple rectangular resistive electrodes for SR monitoring printed on paper: (a) overall SRE, (b) a resistor, (c) contact pads, (d and e) intersection points of SREs fabricated by two different methods [27], copyright 2021 IEEE; (**F**) roll-to-roll-printed electrodes with a comb-like spiral structure integrated in a complex wearable device for sweat collecting and rate monitoring [33], copyright 2023 John Wiley & Sons.

Screen printing represents the most popular approach due to its straightforward setup and manufacturing capability. In general, during the printing process, a viscous ink or paste is transferred through a mesh screen onto a substrate by applying mechanical pressure using a squeegee. The areas of the electrodes are defined thanks to a patterned stencil on the screen. This rapid mask-based method allows for the deposition of well-defined thick layers on various substrates, including flexible materials. This makes it optimal for large-scale, low-cost production of conductive patterns and functional layers in wearable and disposable sensing devices. As displayed in Figure 3A, screen-printed silver electrodes were exploited to fabricate a multi-purpose application wearable sensor [24]. It is worth mentioning that despite the well-known advantages of process simplicity, the overall success in reliably printing effective electrodes strongly depends on critical parameters that govern the fabrication process; these parameters include material characteristics, printing settings, curing methods, and working atmosphere conditions [38]. Consequently, although screen printing is often considered as a straightforward method that requires little-to-no optimization, a thorough examination of the various factors and parameters involved in the fabrication procedures is essential to optimize the process itself and achieve consistent results. Interestingly, Somarathna et al. highlighted this critical aspect by investigating in detail the screen printing process of an SR electrode (SRE) in detail; they systematically addressed different challenges such as poor reproducibility, clogging and non-uniform ink flow encountered when using water-based, carbon-filled conductive ink, ultimately demonstrating the success of the proposed approach [27]. For instance, to prevent screen clogging, the authors relied on optimizing printer modes and time processes and on atmosphere and viscosities modifications; additionally, they examined the crack shaping of the printed resistors and explained that eliminating the rigid polypropylene (PP) from the Thermoplastic Polyurethane (TPU) substrate improved stress allocation, which was determined as a highly efficient technique to prevent resistor fracture. An image of the fabricated resistors is displayed in Figure 3F, which reports two various printing procedures. Therefore, as previously underlined, the authors displayed that robust resistors can be successfully screen-printed with water-based carbon-filled conductive inks by carefully controlling the circumstances of the printing procedure and opting for appropriate materials, printing factors, curing conditions, and preparation process rate.

Analyzing the literature in terms of printed geometries (Figure 3A,C,E,F), typical solutions realized by means of these techniques include circular or rectangular macroscopic electrodes for EDA, with typical dimensions ranging from five to tens of mm [24,27,29,30]; a further identified solution is represented by macroscopic interdigitated electrodes with millimetric thickness of the comb structures, as reported in [28] and displayed in Figure 3D. Although the macroscopic dimensions of traditional screen-printing approaches are advantageous for fast scalability, they represent a critical limitation when high-resolution/high-density electrode arrays are required, such as in EDA mapping. To overcome this limitation, the studies reported in [26,33] successfully demonstrated the fabrication of higher-resolution geometries, achieving a feature size of approximately 80 µm. Furthermore, especially when targeting sweat rate or volume monitoring applications, a promising approach involves the integration of customized microfluidics (e.g., spiral or serpentine patterns) with electrodes specifically designed in geometries that align with sweat flow; this configuration enhances overall sensitivity in response to variations in the measured variables (as displayed in Figure 3B,F).

### 3.2. Non-Contact Printing

As highlighted in the literature, non-contact printing techniques represent a valid alternative to contact printing in EDA and SR electrode fabrication when high throughput is not required and when it is preferable to address other critical aspects, such as improved resolution, highly customizable geometries, and a possibility to print over non-conventional surfaces (e.g., 3D surfaces). Among the various identified techniques belonging to this group, inkjet printing, aerosol jet printing (AJP), and microdispensing were the most employed to fabricate EDA and SR electrodes. Indeed, these nozzle-based methods were reported to provide higher resolution and greater material versatility and to be appropriate for printing materials in a delimited area with high reliability in the deposition. An overview of the different reported methods is provided in Table 2.

Inkjet printing represents one of the most popular methods for fabricating customized SR and EDA structures [30,39,40,41] due to its ability to produce detailed patterns and its greater flexibility. It is a digital technique that allows for precise deposition of small droplets of functional materials onto generally flat substrates. In general, the printing process includes the controlled ejection of ink through multiple nozzles, exploiting thermal or piezoelectric actuation, following a pattern directly uploaded from digital design software. The main advantages of this method include high resolution, reduced material waste, and compatibility with a wide range of substrates. Examples of combinations and/or comparisons between inkjet printing (IJP) with screen printing (SP) are reported in [30,40], focusing on designs with various resolutions and/or with different ink viscosities.

In addition to IJP, more advanced techniques, such as AJP and microdispensing, were also reported for improving EDA and SR sensing devices, particularly in terms of enabling the assembly/integration of electrodes, interconnection, and electronics during the same printing session [42], as well as the ability to print on conformal 3D substrates [36]. The crucial ability of novel non-contact printing techniques to realize not only sensing elements but also integrated structures (e.g., microfluidics and interconnections) in a single process represents a significant and impactful innovation for EDA and SR devices. This approach has the potential to simplify manufacturing, reduce costs, and enhance production scalability—overcoming the limitations of traditional methods, which often depend on expensive processes, highly specialized facilities, cleanroom environments, and often result in high material waste.

Addressing these issues, Kim and coworkers presented a novel approach to fabricate soft bioelectronics, including both electrodes and conditioning systems, using AJP [42,43]. In fact, the AJP method was recognized as a versatile printing technique, enabling creation of high-resolution features as small as 10 μm across a broad range of ink viscosities [44]. The process includes the consecutive printing of different inks, followed by a curing stage. Flexible electrodes were prepared using AJP to create open-mesh-shaped polimmide/graphene layers on a PMMA-coated glass slide. The PMMA was then dissolved in a solvent, allowing the electrode layers to be relocated onto medical-healthcare-grade, class VI silicone tape and connected with the flexible circuit. The printed graphene electrodes preserved their integrity and exhibited a stable resistance alteration when exposed to frequent mechanical strain, proving them to be desirable for on-skin applications. Furthermore, the skin-to-electrode impedance assessment showed that the contact quality of this printed electrode was comparable to that of common Ag, Au, and traditional gel electrodes.

A soft bioelectronic device with printed electrodes and circuits was then fabricated to capture various physiological signals (ECG, EMG, and EDA).

**Table 2:** Non-contact printing techniques-based EDA and sweat monitoring electrodes.

Printing Technique	Inks	Substrate	Flexibility/Stretchability	Electrodes Geometry	Application	Refs
Inkjet printing	silver	polyimide	flexible	interdigitated (teeth thickness 0.1 mm)	sweat volume	[39]
	silver	PET	flexible	rectangular (conductivity: 2 × 0.4 mm, volume: 30 × 0.4 mm)	multi-purpose (conductivity, volume, and copper ion)	[40]
	graphene	polyimide	flexible	square (2.5 mm)	EDA	[41]
	silver	PLA	flexible and stretchable	circular (5 mm)	EDA	[30]
AJP	graphene + polyimide	silicone tape	flexible and stretchable	serpentine-shape pattern (thickness: 0.6 mm equivalent area: 4 cm^2^)	multi-purpose (EDA, ECG, EMG)	[42,43,45]
Microdispensing	silver	high-impact polystyrene (HIPS)	flexible and stretchable	square (2 mm)	EDA	[36]

### 3.3. Three-Dimensional Printing

In addition to contact and non-contact printing techniques, additional manufacturing processes include 3D printing techniques employed for fabricating 3D electrodes or supportive substrates by sequentially depositing layers of material according to specific digital designs. The specific techniques mentioned are 3D direct ink writing (DIW), fused deposition modeling (FDM), and 3D powder bed printing (PBP). It is important to note that 3D printing can serve two distinct roles in sensor fabrication. In some studies, electrodes were directly printed using conductive or polymeric inks (e.g., DIW or conductive FDM, which are presented in Figure 3A, 3B, and 3D, respectively). In others, 3D printing was used to create molds or templates—for example, Figure 3C reports rigid structures fabricated via FDM or PBP—that were subsequently filled or coated with conductive or elastomeric materials to obtain molded electrodes. This distinction is crucial, as the latter are not “printed electrodes” in themselves but molded electrodes fabricated using 3D-printed tools. Studies which adopted these techniques are reported in Table 3.

**Table 3:** Three-dimensional printing techniques used for EDA and sweat monitoring applications.

Printing Technique	Inks	Substrate	Flexibility/Stretchability	Pattern Geometry	Application	Refs
Direct ink writing (DIW)	silicone-based polymeric ink	adhesive layer with predefined inlet holes	flexible stretchable	spiral microfluidic channel is about 78 μL with a circular sweat collection zone (1 mm in diameter)	liquid collection and sweat monitoring	[46]
	silver	polyimide	flexible	serpentine	sweat rate in situ	[47]
	silver and carbon	not specified	flexible	not specified	skin humidity	[48]
	silver	plastic mold	flexible	macroscopic pads and tracks customized depending on mold	EDA	[49]
	silver inks/carbon-based inks/supercapacitive materials	polyimide	flexible	square electrodes	EDA/humidity	[50]
	silver	thermoformed plastic substrate	rigid	square electrodes	EDA in automotive/wearable.	[51]
Light-based 3D multi-material printing.	PEDOT:PSS and mixed materials	various (polyimide/glass/flexible stretchable polymers)	rigid/flexible/stretchable	micro-patterned structures	various physiological signals (EDA)	[52]
Direct FDM printing	PLA + carbon black	PLA	not flexible	circular flat electrodes (15 mm) spherical and conical non-flat electrodes	EDA electrodes	[53]
	metallic printed traces/liquid metal/conductive composites	3D-printed flexible substrate with pneumatic driven electrodes for health monitoring	flexible	3D conformable electrodes, pneumatic-driven	EDA monitoring	[54]
Molding using 3D-printed mold (FDM)	liquid metal injected into acrylonitrile butadiene styrene mold (ABS)	--	not flexible	honeycomb mold	mold for realizing EDA electrodes via injection of liquid metal	[55]
3D-printed flexible substrate (TPU)	TPU	--	flexible, stretchable	4 mm high, 40 mm long, and 22 mm wide module with two circular areas for printing electrodes	substrate for following screen printing/inkjet printing of EDA electrodes	[30]
Molding using 3D-printed scaffold (PBP)	sugar grains	--	not flexible	customized depending on wearable position	mold for realizing 3D multipurpose electrodes by injection of PDMS + SWCNTs	[56]

**Figure 4:**
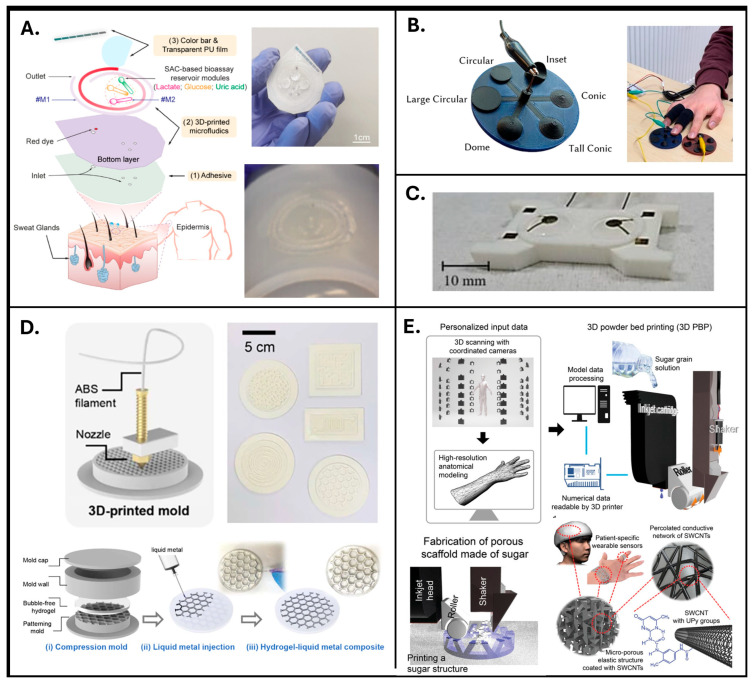
Examples of 3D-printed elements for EDA or SR sensing applications: (**A**) direct ink writing example of fabrication of microfluidics for sweat sensing device [46], copyright 2024 American Chemical Society; (**B**) FDM-printed 3D EDA electrodes with different shapes (flat and non-flat) [53], copyright 2024 ACM, Inc; (**C**) FDM-printed support for following screen or inkjet printing of EDA electrodes [30], copyright 2017 MDPI; (**D**) FDM printing of a mold used as support for following injection of liquid metal for EDA electrode fabrication [55], copyright 2020 American Chemical Society; (**E**) 3D powder-bed-printed porous scaffold for following percolation with hydrogel and conductive elements for fabricating 3D EDA conformal electrodes [56], copyright 2019 John Wiley & Sons.

One of the main challenges facing these printing techniques is the mechanical stiffness of the traditional materials, which can result in electrodes that do not easily conform to the irregular surface of the skin—an issue relevant to both EDA and SR electrodes. Three-dimensionally printable polymers commonly used in fused-deposition-modeling (FDM) 3D printers—such as TPU and polylactic acid (PLA)—present relatively high elastic moduli, making them less suitable for fabricating flexible components with high-resolution somatic structures. To address these limitations, several interesting solutions have been proposed to move beyond rigid 3D printing and to enable the fabrication of flexible and stretchable structures. Schubert et al. [30], for example, proposed geometrically optimized light shapes, including lattice or mesh design, to fabricate a flexible and stretchable membrane that could be deformed under pneumatic excitation (displayed in Figure 4C). As shown in Figure 4D, another solution proposed by Young Choi et al. [55] combined a rigid mold fabricated by FDM with liquid metal molding, enabling the creation of conformal EDA electrodes. Finally, another interesting solution was reported by Ho et al., who presented a novel combination of 3D powder bed printing techniques and soft materials that enables the production of a high-resolution printed framework with flexibility, light weight, and significant conductance [56]. The process displayed in Figure 4E relied on high-resolution 3D scanning to create correct body–structure information, which was then used to print a tailor-made porous scaffold via 3D powder bed printing (3D PBP), exploiting an inkjet head with a spatial resolution of 42 µm. The scaffold was formed from sugar grains of varying sizes, which in fact represents a cheap, eco-friendly, and water-soluble solution, making it ideal for creating a 3D open cellular structure (OCS). Silicone elastomers, such as Ecoflex and PDMS, were then injected into the porous scaffold, which was then dissolved to create a flexible and porous elastomer structure. To impart electrical conductivity, the elastomer surface was coated with a dispersion of single-walled carbon nanotubes (SWCNTs) modified with supramolecular 2-ureido-4[1H] pyrimidinone (UPy) groups to ensure stable dispersion and reduce interfacial energy. This coating created a percolation network that provides electrical conductivity, with a typical resistance of around 4 kΩ and that retains a lightweight density of 0.25 g/cm^3^. This approach allowed for the seamless integration of various materials, such as graphene, silver nanowires, and conductive polymers, which are crucial for achieving high conductivity, biocompatibility, and flexibility. The modular design enabled easy customization, where different biosensors (such as ECG, EMG, and electrodermal sensors) could be added or replaced depending on the application, enhancing the overall device versatility.

In order to have a clear descriptive summary and provide the necessary critical synthesis, Table 4 offers a comparative analysis of the primary printing techniques as previously discussed. As highlighted in Table 4, the optimal printing choice is highly dependent on the target application. For fundamental research and initial clinical prototyping of simple EDA sensors, screen printing remains the most practical method due to its low cost and material versatility. Conversely, for the development of high-performance, complex devices like Strain-Resistive (SR) sensors requiring precise geometries, inkjet printing offers the necessary resolution and digital flexibility. Looking toward scaling-up and future high-volume commercialization, the ultimate transition for flexible EDA and SR sensors will necessitate the use of gravure/flexography (R2R), which—despite high initial costs—offers scalability and throughput essential for consumer electronics and medical devices.

**Table 4:** Comparison of various printing techniques.

Printing Technique	Scalability	Resolution	Relative Cost	Key Advantages	Key Limitations	Best-Suited Applications and Sensing
Screen Printing	High-Volume, Medium	Low (≥50 μm)	Very Low (equipment and materials)	Excellent layer uniformity, lowest capital cost, compatibility with high-viscosity, functional inks, large-area printing.	Poor resolution (limits complex circuits), high material wastage, manual labor often required.	EDA/ECG electrodes, simple interconnects, bulk resistance layers. Ideal for low-cost, disposable sensors requiring large, simple features.
Inkjet Printing	Low-to-Medium	High (≤20 μm)	Medium (high ink cost, low deposition cost)	Excellent high resolution and feature control, minimal material waste, digital, non-contact, multi-material capability.	Limited to low-viscosity, costly nanoparticle inks, potential nozzle clogging, difficult with rough or highly flexible substrates.	SR sensors (complex strain gauges), transistors/micro-circuits, highly detailed sensing elements where precision is paramount.
Gravure/Flexography (R2R)	Very High	Medium (≈20–50 μm)	High (cylinder/plate fabrication)	Highest throughput/scalability (millions of units/hour), superior layer thickness control, robust for volume manufacturing.	High initial tooling cost (non-digital), challenging for quick design changes, limited to specific ink viscosities.	Mass-produced arrays, batteries/power sources, flexible heaters. Optimal for high-volume, low-margin products like flexible displays or simple battery films.
Aerosol Jet Printing	Medium	Very High (≤10 μm)	Very High (equipment)	Prints high-viscosity inks at very high resolution, non-contact, prints on 3D non-planar surfaces, no need for mask/tooling.	Very high equipment cost and complexity, slow relative throughput for large areas, high maintenance requirements.	3D interconnects, multilayer hybrid circuits, precise deposition on non-flat medical devices (e.g., bone implants).
Extrusion/Dispensing (3D Printing)	Low	Low (≥100 μm)	Low–Medium (equipment)	Prints high-aspect ratio structures (thick layers), compatibility with extremely viscous “paste” inks/hydrogels, true 3D geometries.	Slowest throughput, poor lateral resolution, features often require post-processing/curing steps.	Biocompatible hydrogel electrodes, soft EDA electrodes (conforming), complex strain relief structures, personalized medical devices.

## 4. Electrode Materials

Printed electrodes for EDA or SR analysis can be mainly classified into two different classes according to the skin contact model that they implement, including ones that mimic wet electrodes and mainly employ gel-like materials (e.g., hydrogel) and ones that realize dry electrodes exploiting conductive materials (e.g., silver, carbon, conductive polymers), as summarized in Table 5.

### 4.1. Gels and Hydrogels

**Figure 5:**
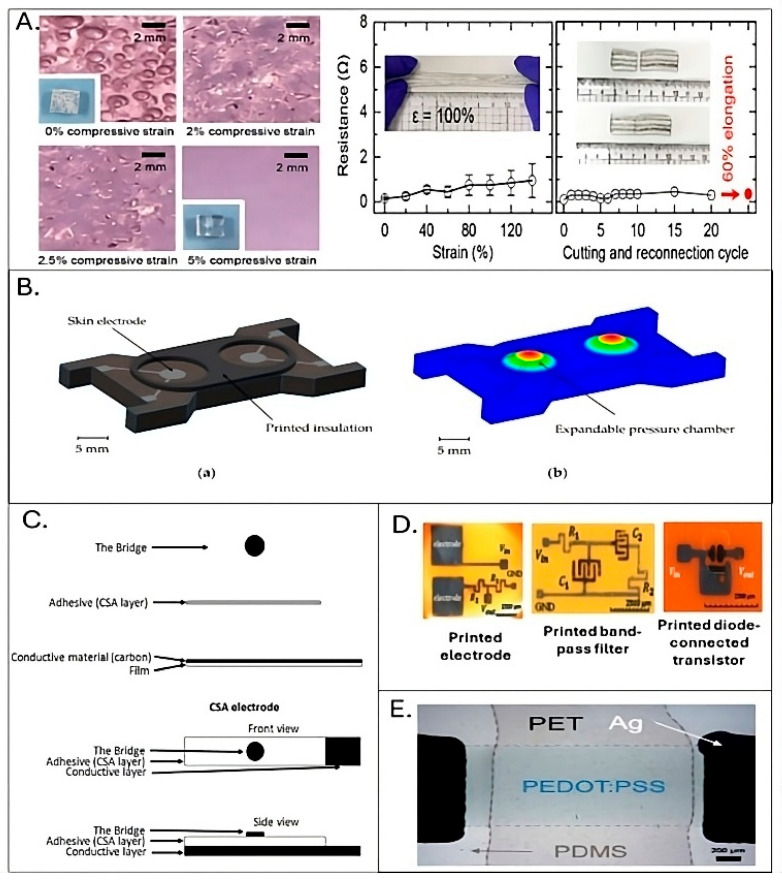
Examples of electrode materials for EDA or SR sensing applications: (**A**) (left) Images of air bubbles in the hydrogel as a function of compressive strain. The inset images are photographs of hydrogel blocks before and after compression; (right) stability of electrical resistance of the hydrogel–liquid metal composite as a function of compressive strain and cutting and reconnection cycle [55], copyright 2020 American Chemical Society. (**B**) (a) Schematic drawing of the electrode module; (b) working principle of the electrode module [30], copyright 2017 MDPI. (**C**) Illustration of the fabrication process of CSA electrodes [59], copyright 2017 Elsevier. (**D**) (left) Printed EDA sensor, (middle) printed band-pass filter for signal processing, (right) printed diode-connected transistor as threshold for stress detection [41], copyright 2022 ACM, Inc. (**E**) Optical microscope image of PEDOT:PSS channel, Ag source/drain electrodes, and PDMS on a PET substrate. Scale bar is 200 µm. Average transfer and output curves of flexible OECTs (*n* = 3) with printed PEDOT:PSS channels, printed Ag source and drain electrodes, PBS gating media, and an activated carbon gate electrode measured while flat and bent [60], copyright 2021 Royal Society of Chemistry 2025.

Hydrogel and gel electrodes are widely used for bioelectrical measurements due to their ability to reduce skin–electrode impedance by maintaining consistent hydration. Their soft, conformable nature ensures improved skin contact, enhancing signal quality in applications like EDA and sweat monitoring. However, important parameters including gel mechanical stability, viscosity, and interaction with the skin over time should also be evaluated. Prolonged use of gels can cause discomfort for users and may alter baseline skin conductance, while gel degradation over time can drastically increase impedance and the overall susceptibility to motion artifacts, ultimately reducing signal quality. These limitations often compromise the suitability of gel-based systems for truly long-term or continuous monitoring via printed platforms [57]. Alternatively, self-healing hydrogels were introduced due to their ability to recover their mechanical and electrical properties after sustaining physical damage [58]. In this regard, Choi and coworkers developed a hydrogel–liquid metal composite for printed sensor applications [55]. The liquid metal electrode pattern was fabricated employing a custom 3D-printed mold. The preparation process for the hydrogel–liquid metal composite involved several key steps. First, a hydrogel solution was made by blending polyvinyl alcohol (PVA), agarose, and borax. The hydrogel was then placed under compressive strain to extract bubbles before being molded using a 3D-printed form. The molded hydrogel was capped with a flat hydrogel layer, joined together via the hydrogel’s self-healing characteristics, creating a patterned structure with empty spaces (shown in Figure 5A). Liquid metal was then injected into these spaces, forming various intricate patterns. The size of the composite pattern is reported to be as small as 1 mm, with the potential for even finer resolution using high-resolution 3D printing techniques. The study specifically examined the electrical resistance of the prepared composite under strain and after self-healing. The composite maintained its electrical stability when stretched up to 140%, with resistance only slightly increasing from 0.1 to 1.7 Ω, indicating the consistent conductivity of the liquid metal electrode. Even after 20 cycles of cutting and reconnection, the resistance remained stable between 0.1 and 0.5 Ω. When stretched to 60% elongation, the resistance stayed within this range, demonstrating the overall durability and self-healing capabilities of the proposed solution. The composite was then used to build a customizable sensor system and tested for detecting biosignals like EMG, ECG, and EDA. Indeed, this unique combination of a self-healing hydrogel with liquid metal is particularly valuable for printed EDA sensors, as it provides excellent conformability, resilience to mechanical stress, and consistently low impedance—crucial factors for capturing subtle skin conductance changes and overcoming the limitations of traditional gel electrodes in flexible devices.

**Table 5:** Materials used for printed electrodes.

Electrode Material	Composed Of	Fabrication Methods	Applications	Refs
Gels and hydrogels	Poly(vinyl alcohol) (PVA), agarose, and sodium tetraborate decahydrate (borax)	3D printing	EMG, ECG, and EDA	[55]
Metals	Silver	3D printing	EDA	[36]
	Silver	IJP and SP	EDA	[30]
	Silver	SP	(ECG, EDA, acceleration, temperature, humidity	[24]
Carbon-based materials	Carbon/salt adhesive (CSA)	SP	EDA	[59]
	Graphene	IJP	EDA	[41]
	PLA and carbon black	3D printing	EDA	[53]
Conductive polymers	PEDOT:PSS	SP	EMG, ECG, and EDA	[29]
	PEDOT:PSS	Screen printing	EDA	[60]

### 4.2. Metals

Metallic electrodes are widely used for biosignal monitoring, including electrodermal activity (EDA) and sweat sensing, due to their excellent electrical conductivity and mechanical stability. Commonly made from materials like silver, gold, or stainless steel, these electrodes provide reliable signal acquisition with minimal impedance, ensuring accurate measurements. They are durable and reusable, making them cost-effective for both research and clinical applications. Additionally, metallic electrodes can be engineered into various shapes and sizes to suit specific applications, owing to their compatibility with various processing methods. For example, in the context of in-mold electronics (IME), Lall et al. [36] emphasizes the utilization of silver-based conductive inks, which provide excellent electrical properties and adhesion to flexible substrates. By adjusting parameters such as ink viscosity, printing speed, and curing temperature, the study achieved enhanced adhesion and conductivity of the silver-based conductive inks. In this work, one key finding was that a precise balance between curing temperature and time improved the ink conductivity while preventing cracking or delamination during the in-mold process. Furthermore, optimized layer thickness reduced electrical resistance and ensured uniformity across the printed circuit. The reported research also demonstrated that fine-tuning the printing speed and nozzle design minimized defects, leading to improved reliability and consistency in the electrodes.

While this kind of electrode typically enhances patient comfort and is ideal for long-term use, this solution faces challenges such as increased electrode-to-skin impedance and vulnerability to movement artifacts [61]. They also fail to achieve the same level of physical junction due to insufficient adherence and conformity to indented surfaces, leading to air-containing space that elevates electrical impedance. Consequently, the impedance recorded with dry electrodes can fluctuate significantly, even when considering minor variations in contact pressure [62]. To overcome such obstacles, Schubert and coworkers presented a novel on-demand electrode–skin contact module designed for future medical care implementation [30]. The module consisted of a polymeric substrate, electrodes, and an insulation layer that keeps the electrodes separated from the skin; when a measurement is required, an extendable pressure chamber is blown up to make contact with the skin and then retracts afterward (shown in Figure 5B). This method provided three main benefits, which were underlined by the authors; in fact, it guaranteed reliable electrical contact, reduced skin irritation by shortening contact duration, and was entirely printable, making it cost-effective, easily scalable, and highly customizable.

Thanks to the versatility and compatibility of metallic electrodes with surface modifications—including coatings with conductive polymers—to further enhance their performance, Alastalo et al. designed a vertically stacked modular skin patch for chest and arm use, which measured both electrocardiogram (ECG) and GSR [24] (as shown in Figure 3A). They highlighted the critical role of advanced electrode materials in enhancing the performance of wearable biosignal monitoring systems. The electrodes in this skin device were specifically crafted using flexible, stretchable, and conductive materials such as silver-based inks, conductive polymers, and carbon-based materials. These materials were chosen for their ability to maintain strong electrical performance while conforming to the skin surface and movements, minimizing contact impedance and enhancing signal accuracy.

Silver-based inks offer high conductivity and stability, making them ideal for capturing low-noise signals. However, since these materials are often prone to oxidation and poor adhesion during prolonged direct contact with the skin, coating them with conductive polymers, such as PEDOT:PSS, has been shown to provide additional advantages in terms of flexibility and biocompatibility, ensuring prolonged wearability without causing irritation. Together, these materials enabled the electrodes to efficiently collect EDA, ECG, and other biosignals with improved precision and comfort.

### 4.3. Carbon-Based Materials

Carbon-based dry electrodes represent a promising choice for printed sensor applications, particularly for EDA, due to their high conductivity, flexibility, and biocompatibility. Materials such as carbon nanotubes (CNTs) and graphene offer stable electrical performance without the need for gels, enhancing comfort and wearability for long-term use. Additionally, their robust mechanical properties make them ideal for integration with flexible systems. However, formulating stable and high-performance printable inks using nanomaterials often presents challenges related to dispersion, aggregation, and rheological control—all of which are critical for achieving consistent electrical properties and high-resolution printed patterns.

In this regard, Posada-Quintero and coworkers showed the effectiveness of a new dry carbon/salt adhesive electrode by comparing it to Ag/AgCl hydrogel electrodes (Figure 5C) [59]. A key advantage highlighted was the ability of these formulations to maintain low impedance (comparable to wet electrodes) while eliminating the shelf-life restrictions inherently associated with hydrogels, offering a significant practical benefit for long-term, ready-to-use printed EDA sensors.

Zhao et al. presented a novel fully printed EDA sensor using graphene material designed to accurately monitor stress levels through skin conductance (Figure 5D) [41]. The study demonstrated that the ability of the full-printed carbonaceous-based sensor to precisely capture electrodermal signals correlated with stress levels during various activities outperforms traditional EDA sensors in terms of signal clarity and ease of use.

Schmitz and coworkers explored the use of 3D-printed electrodes employing a composite material of PLA and carbon black for EDA measurement and examined the effect of electrode shape on measurement precision [53]. In an experiment involving six participants, six 3D-printed electrodes were compared to commercially available nickel electrodes. The findings indicate that these carbon-based printed electrodes were able to accurately measure EDA, with plane-shaped electrodes of a larger diameter demonstrating better performance. Based on their findings, they proposed guidelines for choosing electrode shapes. In particular, to enhance accuracy for electrodermal response, they suggested using electrodes with a larger skin touch area, whereas for low contact resistance, flat-shaped electrodes were identified as ideal. Pointed electrodes were defined as suitable for applications requiring increased sensitivity. For robust performance, especially in scenarios where motion may interfere with EDA recording, inwardly curved electrodes were reported to be optimal due to their stability, high accuracy, and secure grip.

### 4.4. Conductive Polymers

Electrodes fabricated with conductive-polymer-based dry foam were reported to provide EDA measurements that are similar in accuracy to those obtained by using gel-based electrodes; on the other hand, polymer-based solutions offered greater flexibility and durability.

A polymer blend of poly 3,4-ethylenedioxythiophene (PEDOT) has been investigated for its dual ability to conduct both ionic and electronic currents in EDA measurements, as highlighted by Sinha et al. [29]. Conductive polymer electrodes were also reported to be compatible with scalable manufacturing techniques, such as inkjet or screen printing, enabling efficient fabrication on flexible substrates like paper or textiles. Their adaptability to diverse sensor designs makes them a versatile choice for applications in health monitoring, offering reliable data collection with high sensitivity and minimal signal interference. These properties make conductive polymer electrodes a promising and effective solution for implementing wearable sensors, particularly for continuous, non-invasive monitoring. Various methods, however, exist to create highly breathable electronic tools with strain-insensitive conductance. For example, blending conducting polymers with non-conductive ones like polyurethane, PEG, or PDMS was adopted to reduce Young’s modulus and enhance stretchability. In this regard, Hagler et al. fabricated conductive films employing PEDOT: polystyrene sulfonate (PSS) ink on a TPU substrate using screen printing technique [60]. As shown in Figure 5E, these films demonstrated reversible stretchiness, meaning their resistance remained largely unchanged after being stretched and released. To display the applicability of PEDOT films in wearable electronics, they employed PEDOT film as an EDA sensor, which evaluates the skin’s electrical response against stress and emotional stimuli.

## 5. Sweat Collection Methods

For sweat monitoring to be effective, especially when conducted alongside EDA analysis, it is essential to have a precise method for collecting the sweat; however, this requires the design of a comprehensive system that integrates efficient solutions for collecting, transportation, and detecting sweat. In the investigation of the literature, we realized (summarized in Table 6) that traditional sweat collection methods mainly include absorptive patches or capsules stuck on the skin to capture sweat passively. These techniques, although highly efficient for collecting sweat over time, were reported to suffer in terms of real-time tracking and might be uncomfortable, as they included bulky measurement systems.

In order to overcome these limitations, recently, microfluidic devices were introduced to support continuous sweat sampling directly from the skin using patterned channels to conduct sweat flow [63,64]. Additionally, microfluidic-based systems were identified as a possible solution that can be incorporated with flexible and skin-compatible substrates, providing a less intrusive, more pleasant experience for the users [50].

**Table 6:** Sweat collection methods used for sweat monitoring applications.

Sweat Collecting Methods	Material	Geometry	Fabrication Technique	Refs
Absorbing patches	cellulose paper (grade 2)	four-channel pattern	channel was created by ceramic ink printing as a hydrophobic insulator wall on paper	[65]
	cellulose filter paper, grade 1	square structure with 4 mm width and 40 cm length	laser cut	[39]
	Textile	fractal channel structure	laser cut	[26]
	cellulose filter paper, Grade 541	serpentine	laser cut with PDMS printing as a wall for channel	
PDMS microfluidic channel	PDMS	spiral channel	direct ink writing (DIW)	[46]
	poly dimentyl siloxane (PDMS)	spiral with a 0.4 mm depth and a 5 mm diameter depth	photolithography	[66]
	silicone	turbine-like channel	laser cutting	[67]
Other collection methods	thermoplastics of RGD515 and RGD531	square-like chamber	3D printing	[33]
	double-sided microfluidic adhesive tape (3M 9965) and a polyester sheet	spiral	Laser cutting	[20]

### 5.1. Absorbing Patches of Paper-/Textile-Based Materials

To simplify the fabrication process of collecting devices, hydrophilic porous materials like filter paper have gained significant attention due to their affordability and wide availability. As a porous intermediate, paper was reported to rapidly absorb liquids and leverage the capillary pressure differences between dry and moist areas to direct fluids to targeted locations. Because of its ability to wick water, paper-based systems were implemented as reliable skin-attachable devices for continuous sweat sampling.

To this aim, Yokus and coworkers introduced a cost-effective printed impedance sensor that incorporated paper-based microfluidics for tracking SR [28]. The proposed design exploited paper well, facilitating ongoing sweat collection and evaluation when in direct contact with the skin. The authors specifically examined the capillary-pressure-driven movement of a PBS solution through a serpentine-shaped paper microfluidic channel; as the fluid traveled, its velocity decreased due to reduced capillary pressure, causing slower progress over time (see Darcy’s law and the Lucas–Washburn equation). The channel held 82 µL of fluid in about 30 min, with 15 µL collected per channel segment. Real-time sweat rate monitoring was then achieved by tracking admittance changes as the fluid crossed electrode pairs, with admittance spikes indicating fluid passage. The sensor flow rate aligned with typical sweat production, supporting continuous monitoring without limiting fluid wicking.

**Figure 6:**
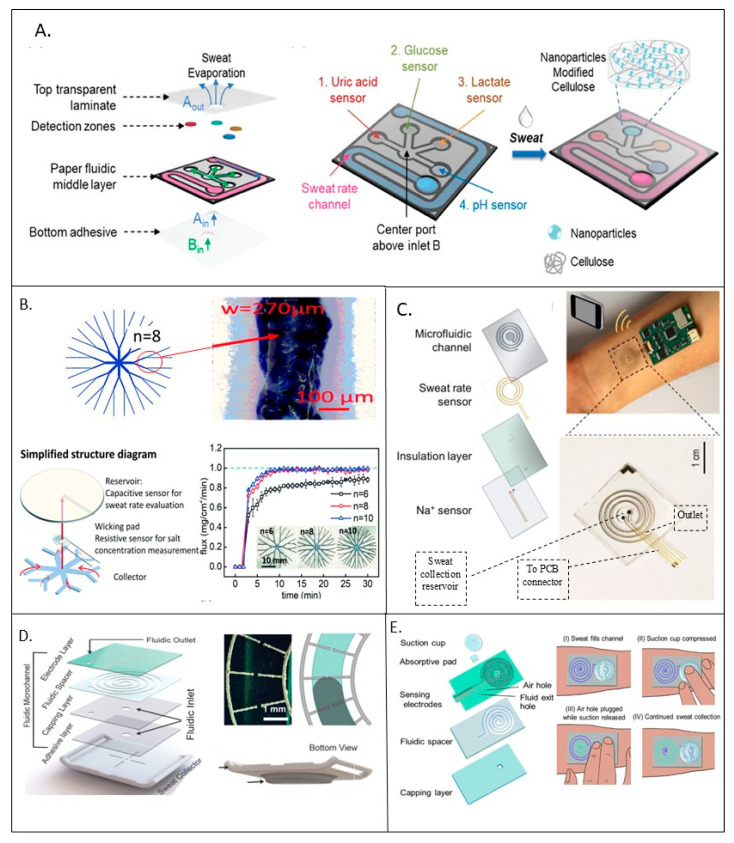
(**A**) (left) An expanded view of the sensor patch and the sweat flow directions in the patch; (right) colorimetric sensor layout and nanoparticle modification of detection zones in the paper middle fluidic layer [65], copyright 2023 John Wiley & Sons; (**B**) (top) schematic framework of the fractal channel structure; (bottom) schematic diagram of the sweat collection/sensing device and collection flux of different fractal collectors under a flux of 1.0 mg cm^−2^ min^−1^ [26], copyright 2021 Royal Society of Chemistry; (**C**) four layers of microfluidic sweat sensor [66], copyright 2018 American Chemical Society; (**D**) layer-by-layer stack of the device structure including and geometry of the face-to-face comb-like electrode—optical image with schematic drawing showing fluid flow in the channel [33], copyright 2023 John Wiley & Sons; (**E**) stack of device layers with reset actuation in four steps [20], copyright 2022 American Chemical Society.

In another study, a colorimetric paper-based fluidic patch was introduced by Zheng et al. for on-site detection of various sweat biomarkers (pH, glucose, lactate, and uric acid) while simultaneously monitoring SR [65]. As depicted in Figure 6A, the sensor patch consisted of three parts: a central paper fluidic layer positioned between a cap transparent adhesive layer and a medical-grade adhesive bed. The central layer included a longitudinal channel with a water-soluble dye for measuring SR and four sensing areas for the detection of different biochemical markers. Fluidic patterns in the central section were printed using ceramic-based ink on cellulose paper to create liquid barriers for sweat flux. Using a syringe tip controlled by an automated three-axis robot dispenser, various patterns were printed on both sides of the paper followed by annealing at 140 °C to ensure smooth, leak-free channels. The simplicity of the color-based detection method was designed to be in agreement with everyday tools, such as smartphones, which have been increasingly used as part of analytical policy. The channel was then modified with moisture-sensitive Co^2+^ ions. The passage of the sweat from the inlet led to a change in the color from blue to pink as the fluid flowed, thus allowing for quantitative fluid flow measurement. Furthermore, the front filling position indicated the stored sweat volume, enabling overall sweat loss calculation.

In another study, textile-based collectors played a transformative role in wearable sweat collection by combining the natural advantages of fabric with the precision of microfluidic technology. Unlike traditional materials like paper, which is faced with challenges in terms of embedding within clothing and that requires additional support structures for wearability, textiles were reported to provide a unique combination of flexibility, breathability, and comfort, making them ideal for prolonged wear. In contrast with paper, which often lacks efficient directional fluid flow, leading to slower response times, textiles naturally wick sweat, guiding it through microchannels formed within fibers or between fabric layers, which was shown to allow for efficient, passive sweat transport from the skin to sensing areas. Additionally, textile-based microfluidics were reported to support integration with soft printed sensors to analyze parameters like ion concentration, pH, and glucose levels, allowing for real-time biochemical assessment during exercise or daily activities [68,69,70]. This approach to sweat collection was not only shown to be able to enhance comfort and user compliance but also enabled large-scale, continuous monitoring without the need for rigid components.

On this basis, a textile-based device with a fractal design was fabricated for quick and efficient sweat collection by Chen and coworkers [26]. The device employed a fast-wicking textile material to improve the absorption rate and reduce the delay time. As displayed in Figure 6B, the sweat collection and detection device consisted of a multilayer microfluidic system; this solution featured a fractal collector at the base, a middle layer containing a flow channel for detection, and a top textile layer serving as a sweat reservoir. The collector directly touched the skin to absorb sweat and channel it to the central reservoir. An interdigital electrode within the main channel monitored the salt concentration via impedance, while a capacitive sensor in the textile reservoir measured the total amount of sweat absorbed. All these parts were made from textiles cut with a laser engraver, and PET films were employed as insulation and to inhibit evaporation. The fractal collector, designed for minimal flow resistance and optimal skin coverage, had eight main branches and demonstrated the fastest induction time (under one minute in high-sweat areas) and high collection efficiency (up to 4.0 μL cm^−2^ min^−1^) without overflow (shown in Figure 6B). It also reported a quick response time (<30 s) for real-time monitoring of SR and salt concentration during vigorous activity. The effectiveness of the proposed solution was confirmed through a running test, offering valuable insights for designing sweat collectors and continuous health monitoring systems.

### 5.2. PDMS Microfluidics

In numerous sweat collection devices made from textiles, hydrogels, and filter paper, significant deviations between real and calculated values due to evaporation still represent a big challenge [71]. Additionally, most sweat collectors lack directional sweat transport, making them undesirable for real-time observation [66]. Polydimethylsiloxane (PDMS) was considered as a powerful alternative due to its unique properties of being highly biocompatible, transparent, flexible, and easy to mold into complex geometries, making it ideal for developing microfluidic systems tailored to wearable sensors. This silicone-based elastomer is inherently hydrophobic, which is useful in the implementation of microfluidic devices, where controlling fluid movement without losing any liquid to the channel walls is a key parameter. For sweat analysis, PDMS-based microfluidic channels are designed to direct sweat toward specific measurement regions or electrodes, where its conductivity or other properties are defined to be monitored. Its transparency allows for easy observation of fluid movement, which is advantageous for real-time monitoring systems. Additionally, PDMS is permeable to gases like oxygen and carbon dioxide, which provides it the inherent ability reduce the likelihood of trapped air bubbles in the microchannels, enhancing device reliability.

To this aim, Chen and coworkers presented a flexible, wearable health-checking device, produced by an innovative one-step continual fabrication technique using 3D printing with direct ink writing (DIW) [46]. This device featured self-supporting spiral microfluidic channels based on hydrophobic silicone-based polymeric material designed to measure sweat flow rate and biomarker contents (depicted in Figure 4A). Theoretically, this spiral-shaped channel enabled collecting sweat for more than 30 min, depending on the exercise intensities. Direct ink writing allowed for the printing of a self-supporting construct that collected sweat, eliminating the requirement for sacrificial foundation materials and, furthermore, resolving adulteration and evaporating concerns, which were identified to be common in conventional sampling process.

Using similar materials, Nyein and coworkers presented a flexible, wearable microfluidic patch designed to capture dynamic sweat secretion and measure the sodium ion (Na^+^) concentration as well as SR [66]. As presented in Figure 6C, the PDMS-based microfluidic system supported close skin contact, accurately transferring sweat through a spiral structure that minimizes interference while ensuring continuous flow. Na^+^ sensors at the sweat collection chamber monitored the ionic concentration as sweat accumulated, while an impedance-based sensor measured SR by tracking the decrease in resistance as sweat filled the channel. The device featured a compact, commercially available printed circuit board (PCB)-integrated design, which divided signal paths to guarantee precision in simultaneous Na^+^ and SR monitoring.

### 5.3. Other Collection Methods

The scientific literature has reported several further sweat collection methods. For instance, Dautta et al. also described an adhesion-free, wearable sweat-sensing device that offered an easy, non-intrusive, and precise way to monitor sweat levels during extended exercise sessions without needing external assistance [33]. The device mainly consisted of two parts: (1) a sweat chamber and (2) a fluidic microchannel. As illustrated in Figure 6D, these two components were connected by an adhesive layer that allowed sweat to move between them through a common inlet. The fluidic microchannel part comprised a capping layer, a fluidic layer, and an electrode layer. The top layer featured comb-like silver electrodes printed using roll-to-roll (R2R) rotary screen printing on a PET substrate, which allowed for high-efficiency fabrication and consistency across devices. A laser-cut double-sided tape defined the fluidic passage, which was sealed with a hydrophilic covering layer to control sweat wicking. This design ensured that sweat travelled smoothly from the collector to the spiral microfluidic channel, facilitating reliable admittance measurements from the electrodes. The layered structure simplified manufacturing and minimized fabrication errors, leading to consistent sweat measurements.

The small volume capacity of most wearable SREs was reported to limit, in general, their practicality for real-world applications. For example, a microfluidic collector with a 4 cm^2^ area and a 24 μL channel volume was reported to be able to only track SR for around 8 min during exercise before reaching its capacity, necessitating frequent patch replacements [35]. This limited measurement period prevented the sensor from effectively monitoring significant changes in hydration over time, making it unsuitable for continuous, autonomous use. A cursory technique to overcome this issue was proposed by Barya et al. [20]; in fact, the authors reported a resettable SR sensor that was engineered for constant and precise SR monitoring during intense activity. The proposed device was produced using a high-throughput roll-to-roll (R2R) method, allowing for large-scale manufacturing with minimal irregularity. As shown in Figure 6E, the sensor featured a microfluidic path with integrated electrodes and a PDMS reset key. The device was constructed from mass-produced layers, including a laser-cut separator layer that shapes the path, a polyester sheet with an entry for sweat, and a PET layer with screen-printed electrodes to track SR. Sweat was planned to travel through a spiral microchannel and exit through a designated hole. The channel specifically had a capacity of up to 10 μL before needing a reset, which was achieved by pressing the PDMS key to clear the channel, using either negative or positive pressure methods. The flow rate monitoring in this sensor was achieved using two separate comb-like electrodes with interdigitated spokes that extended into a spiraling fluid path. As fluid moved into the channel, it met these electrode rods in sequence without touching the main electrode backbones. Unlike previous designs that measured continuous admittance alteration, this sensor detected discrete admittance steps when sweat reached and connected the next electrode spoke. These steps were linked to specific sweat volumes, allowing for accurate sweat flow rate measurement. The spacing of the electrode spokes provided in particular a volumetric resolution of 72 nL, enabling precise tracking of typical SR during exercise.

In another study, Yang and coworkers presented an all-printed wearable system for sweat volume/rate measuring and sensing copper levels [40]. The device consisted of four distinct layers, each with specific functions. The first layer, fabricated using bioadhesive tape, connected with the skin and was designed to enable interaction with reverse iontophoresis electrodes and collect sweat. The second sheet, prepared with PET, featured inkjet-printed reverse iontophoresis electrodes on one side and screen-printed copper detection electrodes on the other. The next layer, also made from bioadhesive tape, included a microfluidic chamber while leaving the electrode contacts exposed. The last sheet, which was an additional PET layer, was printed with conductivity and volume sensors, and it sealed the microfluidic channel. A hole at the end of this layer allowed sweat to depart and evaporate via a paper-based sponge, enabling the device to reset for reuse. Through this simplified design, they managed to address typical issues, including sample extraction, both continuous and in real time.

## 6. Measurements Methods

Printed sensors for EDA and sweat monitoring rely on two key measurement methods: DC conductivity/resistance and AC impedance/admittance. In general, DC measurements involve applying a constant voltage or current across electrodes to assess changes in resistance or conductivity, which vary based on sweat presence and electrolyte concentration, making this method useful for tracking SR and skin hydration. In contrast, AC impedance/admittance measurements use alternating current at different frequencies to analyze the skin–sweat interface, providing deeper insights into sweat composition and hydration dynamics while reducing signal artifacts.

Although similar DC or AC measurement strategies are typically implemented for both EDA and sweat monitoring, the sensing parameters targeted are usually different. Focusing on EDA, the sensing parameters measured typically include one or more skin electrical properties, depending on the measurement setup; these mainly comprise skin conductance (G), skin resistance (R), skin impedance (Z), and skin admittance (Y). Skin conductance, measured in microsiemens (µS), is one of the parameters most commonly employed in psychophysiological studies; it directly quantifies how easily an electrical current passes through the skin and is highly sensitive to changes in sweat secretion. Skin resistance, the inverse of conductance and measured in ohms (Ω), provides equivalent information but is rarely reported in the literature due to its nonlinear response to changes in local perspiration. Skin impedance is a complex metrological quantity—comprising both real and imaginary parts—that varies with frequency and mainly includes resistance and capacitive components; it is useful not only for evaluating sweat volume but also for bioimpedance analysis, which specifically addresses the composition of skin layers. Finally, skin admittance, the reciprocal of impedance, is a measurement parameter often preferred in systems that aim to emphasize capacitive and dielectric properties of the skin while reducing polarization artifacts, such as wearable devices. Regarding SR, the targeted sensing parameters typically include sweat volume and SR, while specific electrolyte composition is usually addressed in clinical setting. All these parameters—as already mentioned in Section 5—can be computed only by comparing acquired values of resistance/impedance with appropriate calibration curves obtained during preliminary laboratory characterization. Furthermore, printed sensing solutions for both EDA and SR may rely on the use of either DC signals (for resistance or conductance) or AC signals (for impedance or admittance), depending on their design, electronics complexity, and intended application (e.g., stress monitoring, hydration assessment, biofeedback). The optimal selection and analysis of these parameters are fundamental for achieving accurate and reliable EDA monitoring, in particular when real-time approaches are required. In the following paragraphs, DC and AC measurements methods are described and analyzed in detail, with reference to the papers included in this review; this includes a specific description of the metrological features extracted from the defined parameters in order to enable comparison with other studies.

Table 7 summarizes the main applications and metrics that can be found associated with each of the previously mentioned methods. In addition to these main categories of properly electrical measurements, in particular for SR monitoring, printed colorimetric and surface plasmon resonance devices were also often proposed as alternative or complimentary methods, as displayed in Figure 5E [25,46,65]. By integrating a combination of these approaches, printed sensors are reported to enhance the accuracy and reliability of wearable devices for continuous physiological monitoring.

### 6.1. Conductivity/Resistance Measurements

In the electrical investigation of skin, the measurement of skin resistance or, reversely, of its conductivity represents one of the easiest measurement techniques employed to provide valuable insights into physiological and psychological states. Thus, they reflect the ease with which electrical charges move through the skin, primarily influenced by the presence of sweat and ions. Resistance measurement typically employs a constant direct current (DC) to ascertain the voltage drop across the skin, allowing for the calculation of resistance based on Ohm’s law (R = V/I). Conductivity measurement can be performed by exploiting resistance measurements and then calculating the inverse of the results obtained via proper conductometers. Conductometers employing DC exosomatic measuring instruments apply a DC voltage to the skin via electrodes. By measuring the ratio of the applied voltage and resulting current I, the skin conductance G is calculated (G = I/V). All these methods are sensitive to changes in skin hydration levels; increased sweat production during stress or arousal leads to higher conductivity and lower resistance. While direct conductivity measurements can offer a more rapid response proportional to variation in skin state, resistance measurements are often easier to implement in terms of front-end electronics. All the techniques mentioned can be found employed for all applications related to EDA and also, in some cases, for SR monitoring. The most significant also include, in addition to the traditional EDA targeting stress monitoring, EDA related to biofeedback, hydration monitoring, and the assessment of dermatological conditions.

The main metrological parameters extracted from measurement analysis for defining the performances of the electrodes can be observed as the difference between EDA and SR applications.

Regarding EDA, a wide variety of metrological parameters are usually extracted from the raw signals associated with either skin conductance (G) or skin resistance (R). Common signal processing techniques are typically employed to remove motion artifacts and interferences and to better emphasize the frequency components characteristic of EDA and SR measurements while suppressing unwanted ones. In general, these strategies involve the use of pass-band and low-pass filters—including smoothing techniques such as moving average and median filters—as well as signal normalization. The main features extracted from the processed signals mainly include average G or R values over defined time windows, averaged root mean square (RMS) values, and the number of peaks detected within specified times intervals.

An example of the use of RMS can be found in [42], where quantifying the variation in the RMS of the EDA signal, variations in sleep stages were detected, with fewer peaks during REM sleep and more as sleep deepened. The reported findings closely matched prior research, with the two-epoch window showing the highest significance in differentiating sleep stages. Despite filtering traditionally being performed offline or online by exploiting traditional microcontroller, an interesting novel perspective was proposed by Zhao et al. [41], where low-pass filters were directly printed next to EDA electrodes as interdigitated capacitive electrodes.

In particular, when developing new printed EDA sensors, one of the most critical aspects concerning measurement is the validation against a proper reference/gold standard. Most frequently, the references used in the literature are commercially available electrodes [53]. The most employed strategy includes placing customized and commercial electrodes on a nearby site simultaneously, acquiring signals during the same task, and then assessing correlations to obtain a similarity metric between the skin conductance level (SCL) of both signals, the accuracy through precision, defined as how many of the detected skin conductance responses (SCRs) were correct, and finally recall, defined as how many SCRs that occurred were detected. These parameters are reported to be then used to calculate specific indexes, for example F1, which is useful to compare different electrode types. Other interesting features that can be found quantified to compare printed electrodes are shape properties of the EDA signal, such as the peak conductance and rise time, i.e., the time between stimulus and peak conductance. However, since these properties depend on many factors that vary depending on the person under test, how often they were exposed to the testing stimulus, and how well the electrode contacted the skin, most frequently, inter-subject analyses were performed, relying on peak quantification rather than shape-based classifiers [60]. Concerning SR sensing, despite the fact that the most employed measuring technique involves the use of AC signals (see the next section), DC resistance/conductivity measurements can be found as useful simpler alternatives [27]. Digital multimeters are usually employed with high accuracy (6–7 digits). In order to assess the effect of the surrounding environment on the ink characteristics that can affect resistance, it is of fundamental importance to characterize the electrodes in term of sensitivity and linearity when quantifying different amounts of liquids in different thermal and humidity conditions by exploiting controlled climatic chambers.

Regarding front-end electronics made for these measurements, interesting strategies can be found to make this measurement portable and unobtrusive for patients. For example, Kim et al. [42] proposed a solution addressing wearable bioelectronics and proved that it was a novel and effective tool for sleep stage classification at home, offering improved flexibility and form factor compared to previous devices.

### 6.2. Impedance/Admittance Measurements

Impedance or admittance monitoring represent the predominant strategy that is reported in the literature that is employed in printed SR and volume sensors. In fact, both these properties can be quantified by imposing alternating (AC) currents or voltages at a single frequency or across a range of frequencies (spectroscopy) on the skin to provide feedback not only in terms of the resistive but also of the capacitive properties. This technique can provide comprehensive information regarding the skin response, sensing narrower physiological alterations than simpler DC resistance.

A critical challenge in developing printed EDA sensors is achieving consistently low electrode-to-skin impedance. In fact, this criterion directly relates to signal quality and noise reduction, particularly for low-frequency signals (<100 Hz) that characterize the EDA frequency range. In this direction, Jang et al. successfully demonstrated unobtrusive ambulatory sensing using graphene e-tattoos (GET). In fact, they directly acquired signals from the palm of the hand—an optimal location for EDA measurement—by utilizing sub-micron-thin printed graphene electrodes [73]. Specifically, they resolved the challenge of interfacing these flexible devices with rigid data acquisition systems through designing heterogeneous serpentine ribbons (HSPRs). This novel printed structure, which involved partially overlapping graphene and gold serpentine traces without using adhesive, was fundamental for obtaining a remarkable fifty-fold reduction in strain concentration at the rigid–flexible interface. This mechanoelectrical robustness represents a key factor to maintain long-term electrode stability and signal consistency, even during movement. By combining the GET + HSPR architecture with a soft interlayer between the tattoo and a commercial EDA wristband, the authors achieved a fully wearable and unobtrusive EDA monitoring solution. Based on these results and analyzing recent comparative data, an ideal impedance target for printed EDA electrodes can be reasonably identified in the range of 100–500 kΩ at frequencies below 100 Hz. Indeed, these values could serve as a preliminary benchmark for future designs.

The main metrological parameters that are quantified and exploited for comparison between printed SR sensors are sensitivity, usually defined as the slope of the calibration curve obtained when exposing the sensor to controlled volumes of liquid, and the limit of detection, defined as the admittance value corresponding to 3 times the standard deviation of dry conditions [67]. To compute the rate in addition to the volume, it is necessary to combine information about the timing and volume. Usually, admittance steps corresponding to discrete liquid additions are identified through post-processing analyses, and this sequence is then converted to a fill volume profile over time using the known volumetric increment of a channel segment. Finally, a discrete flow rate measurement is generated by taking the discrete derivative of this fill volume profile.

Despite impedance methods offering additional information with respect to DC measurements, variations in sweat salinity, motion, and environmental condition changes (e.g., temperature and humidity) can still affect measuring precision. Interesting approaches can be found among the literature that are focused on the possibility of combining admittance/impedance measurements with other sensors or with more sophisticated signal analysis to address this issue. Regarding the first approach, the combination of impedance measurement targeting sweat volume with temperature or ions sensing represents an interesting approach. For example, Tonello et al., in [39,74], proposed integrated devices that could integrate SRE with inkjet-printed sensors that are able to quantify potassium and sense temperature. To determine SR sensing via the impedance spectroscopy method, an in vitro test was carried out with a spongy patch simulating human skin, with interdigitated electrodes placed beneath it. Known volumes of sweat were sequentially added, and impedance measurements were performed. The results depicted the impedance magnitude at 10 kHz relative to changes in sweat volume. The experiment was based on typical SRs for each person at rest and during exercise using sweat volumes ranging from 10 μL/cm^2^ to 200 μL/cm^2^ in increments of 10 μL/cm^2^, resulting in a higher sensitivity of 0.6 kΩ/(μL/cm^2^), which was obtained for lower volumes of sweat.

Moreover, calibration tests confirmed the device’s ability to simultaneously monitor potassium ion quantity with a sensitivity of 40 mV/decade and temperature with a sensitivity of 0.0384 Ω/°C and an accuracy of 11.93%. These results lay the groundwork for future in situ applications of the multisensory wearable bracelet.

Another interesting approach to increase the robustness of sweat volume and rate monitoring was proposed by Wei and coworkers in [67]; they specifically presented an integrated microfluidic patch for simultaneous SR and chloride concentration sensing, and they additionally exploited pulse-style signals triggered by specific sensing events as a processing strategy to reduce noise interference. The patch featured a “sandwich” structure with electrodes placed above and below the microchannel. Sweat triggered high-admittance pulses as it reached specific points in the channel, improving measurement accuracy and reducing noise interference. The section view of the SR sensor microchannel showed that narrow upper electrodes defined trigger sites where sweat could reach both the electrodes, forming a current path. An equivalent circuit model explained the conduction mechanism at these sites, involving capacitance, resistance, and impedance. In fact, as sweat moved through the channel, it created a stepped increase in total admittance when it reached a trigger site, producing an “admittance pulse”; indeed, this pulse acted like a stopwatch, marking the time when the sweat reached each site, allowing for precise tracking of sweat movement through the microchannel. Additionally, an interdigital electrode was incorporated for chloride concentration detection. The patch, which was small and flexible, could be easily attached to various body parts, including the forearm, forehead, or back, and it integrated with a flexible printed circuit board (FPCB) for reliable sweat collection and data acquisition.

## 7. Discussion on Challenges and Future Directions: The Need for Standardized Benchmarking for Performance Comparison

Despite the detailed information provided by the reviewed studies regarding sensor design, fabrication processes, and integration, only limited knowledge is available concerning quantitative performance metrics that can enable direct comparisons among the different solutions. Nevertheless, several significant contributions present in the analyzed literature can be highlighted as relevant to outline the current state of the art and highlight the main achievements in this field.

Regarding EDA, Kim et al. [42] developed one of the most accurate and stable solutions, based on a flexible printed system capable of monitoring multiple parameters. Their flexible solution—which was specifically based on graphene electrodes realized via AJP technology—demonstrated stable electrical resistance under mechanical strain while maintaining impedance values comparable to those of commercial Ag/AgCl electrodes. This sensor performance enabled the realization of an accurate sleep stage classification through RMS analysis of the EDA signal. Similarly, Zhao et al. [41] reported a fully printed graphene-based EDA sensors that outperforms traditional hydrogel-based ones in terms of selectivity and signal-to-noise ratio, achieving over 95% accuracy in detecting stress-related events. Schmitz et al. [53] presented an innovative example of 3D-printed dry electrodes with non-conventional shapes, demonstrating that customized electrodes with larger skin contact areas could provide greater stability and precision in EDA signal acquisition compared to commercial nickel electrodes.

Concerning SR and volume monitoring, Wei et al. [67] achieved the highest resolution among the reviewed AC-based sensors by using pulse-triggered admittance steps to track sweat progression through a microfluidic channel. Their design and measurement strategy—based on admittance measurement—represented the key element enabling a high volumetric resolution (72 nL) and optimal robustness against environmental noise. Somarathna et al. [27] proposed a screen-printed carbon-resistor-based sensor that achieved a sensitivity of 0.15 Ω/μL for sweat monitoring, demonstrating reliable performance even under repeated mechanical deformation. This result appears particularly promising for applications where sensors need to be integrated into wearable solutions.

Regarding multimodal systems that integrate sweat sensing with additional physiological monitoring, Nyein et al. [66] presented a flexible patch capable of simultaneously tracking SR and Na^+^ ion concentration. Their system demonstrated reliable performance when validated against gold-standard methods for sweat volume analysis in controlled settings. Furthermore, the hybrid screen- and inkjet-printed wearable platform proposed by Yang et al. [40] achieved good integration and durability by combining reverse iontophoresis, sweat volume measurement, and copper ion detection. Their system was fully resettable and demonstrated the feasibility of continuous, prolonged use, even without the need for adhesives to maintain proper skin contact. Finally, the work of Alastalo et al. [24] demonstrated the scalability and integration potential enabled by printed electronics through its focus on modular platforms for multi-biosignal measurements (e.g., ECG, EDA, temperature).

The rapid progress in printed wearable sensors, as documented in this review, has nevertheless exposed fundamental challenges that currently limit their reliable clinical and commercial adoption. One of the most significant obstacles to advancing the field is the lack of standardized performance benchmarking and rigorous reporting protocols across the literature. As evidenced by the difficulty in realizing a comprehensive comparison in terms of performance (Section 5.2), key metrics—such as long-term stability (under conditions of continuous mechanical flexing and environmental exposure), dynamic range, and sensor-to-sensor reproducibility—are often reported qualitatively or omitted entirely. For these reasons, future research must move beyond mere proof-of-concept implementation by adopting standardized international testing protocols, using a defined correlation methodology against commercially available sensors (i.e., the “gold standard”) and reporting device failure mechanisms and batch-to-batch variation. Achieving consensus on these benchmarking standards is crucial for establishing technological readiness levels and enabling meaningful, reproducible comparisons necessary to translate printed sensors from the lab to real-world applications.

## 8. Conclusions

The performed analysis of the literature highlighted the advancement in the field of printed electrodes focused on EDA and SR monitoring, which represents an outstanding improvement in the area of wearable health monitoring. Their potential to provide real-time, continuous, and non-invasive physiological information makes them crucial players to progress in individual and personalized healthcare, with additional applications in occupational settings. The cost-effectiveness, versatility, and process flexibility of the printing approach exploited for their fabrication stand as beneficial aspects for large-scale applications, including stress, hydration, and monitoring various biometrics. Furthermore, compared to commercially available electrodes, they can be considered a more comfortable and convenient substitute, which can limit risks of irritation and enable long-term use. The relevance of the proposed systematic review lies in the exploration of the current state of the art concerning printed electrodes for EDA and SR measurement, with a particular emphasis on design fundamentals, selected materials, and specific types of printing techniques implemented, as well as on challenges and innovative technologies and suggested paths to improve crucial aspects such as such as signal noise, electrode deterioration, and variability in skin contact.

In particular, the designs identified in most of the reviewed literature addressed flexible, stretchable substrates (e.g., TPU, PET) with geometries ranging from circular-shape electrodes (5–8 mm) to interdigitated comb structures (0.1–3 mm) to obtain enhanced sensitivity. Focusing on the materials, screen-printed silver, graphene-doped carbon, and PEDOT:PSS inks represent commonly selected solutions due to the conductivity and compatibility with skin. The different fabrication processes relied heavily on contact printing, such as screen printing (80 µm resolution) and roll-to-roll methods, and on direct writing and 3D printing, often employed to combine fabrication of electrodes and substrates in the same printing session. Non-contact printing (e.g., inkjet, AJP) appears to be less employed, restricted to specific uses in which higher resolution (20 µm) and complex microfluidic integration is required. Shifting the focus towards the main measurement methods, skin conductance/resistance was generally used for EDA, whereas impedance/volumetric tracking was applied for SR, often combined with custom microfluidic solutions (e.g., spiral-shaped or serpentine channels) to improve real-time accuracy.

By exploiting specific printing techniques—such as 3D printing and AJP—in addition to more commonly used methods like screen or inkjet printing, it may also be possible to directly realize electrodes on 3D surfaces or on top of finished objects (including clothing). This approach can enhance comfort and optimize electrode placement, thereby increasing the signal-to-noise ratio. Relying on open-ended printing processes could also offer advantages in terms of ease of fabrication for hybrid systems that integrate EDA and SR with other sensing elements—such as those used for sweat qualitative analysis or for monitoring other physiological signals (e.g., ECG, EMG, EEG)—which have been highlighted in the recent literature as promising for holistic health monitoring. Moreover, the ability to modify electrode geometry to accommodate bending and stretching, for example, through serpentine or fractal-like patterns, can significantly improve the signal-to-noise ratio by reducing motion artifacts caused by temporary loss of skin contact during movement.

Looking ahead, emerging research envisions a new generation of printed and hybrid electrode architectures that combine the scalability of additive manufacturing with the conformability of ultrathin and bioinspired materials. Notably, graphene e-tattoos with serpentine microstructures [73] have demonstrated ultrathin palm-based electrodes capable of continuous and unobtrusive EDA monitoring with high stretchability and mechanical robustness. Similarly, microfluidic and permeable electrode systems [19] and laser-engraved or inkjet-printed nanocomposites [75] have highlighted how printed technologies can yield breathable, adaptive interfaces that maintain low impedance and signal stability, even during movement. In parallel, bioinspired microfluidic sweat patches for multiday sampling [35] and platforms capable of multiplexed hormone detection [76] demonstrated the integration of chemical and electrical sensing into single printed devices. The increasing use of sustainable, recyclable materials and self-healing printable inks further opens new opportunities for durable and environmentally responsible bioelectronics.

Despite all these innovations, robust and long-term performance of these sensors in real-world conditions could be achieved by overcoming several key challenges. These mainly include material degradation due to mechanical stress, poor signal-to-noise ratio resulting from motion artifacts during real-case scenario monitoring, and the lack of standardized testing protocols for comparing sensor performance across studies. Promising paths to overcome these issues have been recently highlighted and represent an interesting open research path. They include the use of self-healing [77] or crosslinked conductive [78] inks to enhance mechanical durability. Furthermore, plasma treatments or surface functionalization have been proven to improve ink adhesion on flexible substrates [79]. The integration of soft encapsulation layers was reliably used to reduce noise and preserve electrode–skin contact [80]. Additionally, the establishment of unified benchmarking procedures, including impedance targets and accelerated aging tests, will be essential to translate printed EDA sensors from laboratory prototypes to real-world applications and clinically reliable wearable systems [81].

In conclusion, this review proposes a critical and comprehensive analysis of the current potential of EDA and sweat rate/volume sensors, exploring ongoing challenges and promising directions for their integration into next-generation health monitoring systems. Taking advantage of the latest innovations, this work is meant to serve as a key reference for researchers aiming to advance wearable sensor technologies with the objective of enabling a new era of personalized and preventive healthcare. Indeed, recent advancements [82] demonstrate how soft microfluidics and multiplexed electrochemical interfaces represent powerful tools for real-time biochemical and flow monitoring. Considering the precision-medicine paradigm outlined in [16,17] and the validation frameworks proposed in [17], we can strongly state that the next research frontier in this topic will be the standardization of printed sensing platforms capable of quantitatively linking sweat-rate, gland activity, and biochemical signatures.

## Figures and Tables

**Table 7:** Applied measuring methods for EDA and SR monitoring.

Printing Technique	Measured Quantities	Applications	Metrological Parameters	Refs
DC measurements	Conductance (µS-mS) Potential (mV) Resistance (Ω)	EDA	Average SCL; RMS value; number of SCR peaks above threshold	[24,36,41,42,53,60]
		SR/volume	Differential signals with respect to baseline; % changes in resistance	[27,33]
AC measurements	Admittance (mS) Impedance (Ω)	EDA/skin impedance/bioimpedance	Normalized signal; frequency content; number of peaks above threshold;	[29,30,72]
		SR/volume	absolute and differential admittance in time frames	[20,26,39,67]

## Data Availability

No new data were created or analyzed in this study. Data sharing is not applicable to this article.
